# Polyvinylpyrrolidone-Capped Silver Nanoparticles for Highly Sensitive and Selective Optical Fiber-Based Ammonium Sensor

**DOI:** 10.3390/nano12193373

**Published:** 2022-09-27

**Authors:** Revati P. Potdar, Yogesh B. Khollam, Shoyebmohamad. F. Shaikh, Pravin S. More, Abu ul Hassan S. Rana

**Affiliations:** 1 Nanomaterials Application Laboratory, The Institute of Science, Dr. Homi Bhabha State University, Mumbai 400032, India; 2 Research Centre in Physics, Department of Physics, Baburaoji Gholap College, Sangvi, Pune 411027, India; 3 Department of Chemistry, College of Science, Bld-5, King Saud University, Riyadh 11451, Saudi Arabia; 4 Department of Electrical and Electronic Engineering, The University of Melbourne, Parkville, VIC 3010, Australia; 5 Department of Obstetrics and Gynecology, The University of Melbourne, Parkville, VIC 3010, Australia

**Keywords:** optical fiber-based sensor, ammonium sensor, PVP, silver nanoparticles, polyol synthesis, cold synthesis

## Abstract

Herein, aqueous ammonium sensing characteristics of polyvinylpyrrolidone (PVP) capped silver nanoparticles (Ag-NPs) coated optical fiber-based sensors are presented. The PVP-capped Ag-NPs were prepared using cold and modified polyol synthesis methods. Aqueous ammonium detection was carried out by the surface plasmon resonance (SPR) effect observed in the Ag-NPs coated optical fiber system. The effect of cold and modified polyol synthesis methods on optical sensing performance was studied. The optical fiber cladding was modified with PVP-capped Ag-NPs according to the standard protocol for sensing investigation. The probe sensing response was analyzed for varying concentrations of ammonium ions on red, green, and blue LEDs. The sensor characteristics, viz., sensing response, repeatability, calibration curve, and ambient light effect, were investigated for PVP capped Ag-NPs coated optical fiber-based sensor. The PVP capped Ag-NPs synthesized via the polyol synthesis method showed a detection limit of 48.9 mM, 1.33 mV/M sensitivity, and an excellent linear relationship (R^2^ = 0.9992) between voltage and ammonium ion concentration in the range of 0.054–13.4 M concentration. On the other hand, PVP capped Ag-NPs synthesized using the cold synthesis method showed a detection limit of 159.4 mM, a sensitivity of 0.06 mV/M, and a poor linear relationship (R^2^ = 0.4588) between voltage and ammonium ion concentration in the range of 0.054–13.4 M concentration. The results indicate that the PVP-capped Ag-NPs synthesized using the polyol synthesis method exhibit enhanced ammonium ion sensing compared to the cold synthesis method.

## 1. Introduction

Over the past few decades, ammonium produced via nitrogen fixation, decomposition of organic compounds, natural exchange of atmospheric gases, and an ionic compound has been essential in agricultural applications. Further, ammonium has been utilized in many applications, including the pharmaceutical industry, fertilizers manufacturing, production of explosives, and plastics manufacturing. Ammonium is used in fertilizer and is part of many nitrogen-containing compounds. Thus, it finds its way naturally into environmental water. According to WHO guidelines for drinking water quality, the average ammonium ion concentration in water is 18 ppm. The excess ammonium ions intake in the human body can cause various health issues, such as increased blood pressure, leading to severe problems like kidney damage, nervous system problems, etc. [1]. However, at the same time, it is also a significant source of nitrogen for many plant species, particularly those growing in hypoxic soils. It is toxic to most crop species and rarely used as a nitrogen source. Considering the significance of aqueous ammonia, substantial research has been carried out for ammonium ion sensing using various techniques such as ion-selective electrodes, field-effect transistors, spectrophotometric methods, potentiometric measurements, fluorescence detection methods, etc. [2,3,4,5].

Recently, efforts have been made to develop fiber optical-based sensing applications using SPR properties of noble metal nanoparticles. In SPR, light with a specific frequency falls on the metal, which causes the oscillation of free electrons in the conduction band [6]. Thus, it is an optical interaction phenomenon (based on quantum mechanics) effective in monitoring the refractive index-related optical changes in the sensor’s environment [7]. SPR-based applications are utilized in various fields like chemical sensing, bio-imaging, environmental monitoring, glucose monitoring, medical diagnostics, early disease detection, and telemedicine [8,9,10,11,12,13]. Optical fiber sensing is more popular because it has high sensitivity, electrical and chemical passive nature, and immunity from electromagnetic interference over other methods [14].

In the present work, the cladding modification of the optical fiber was performed using PVP-capped Ag-NPs for the aqueous ammonium sensing application. PVP-capped nanoparticles are reported to have excellent selectivity for detecting ammonium ions [15]. PVP-capped Ag-NPs were prepared and characterized. The PVP-capped Ag-NPs modified optical fiber sensor was investigated for different ammonium in concentrations. The sensor tested various light sources, including blue, red, and green LEDs. The various sensor characteristics were investigated, viz., repeatability and calibration curves. Finally, a sensing mechanism was proposed for ammonium ion sensing.

## 2. Materials and Methods

Acetone, ethylene glycol, silver nitrate, ammonium solution, potassium chloride, polyvinylpyrrolidone (K 90), dichloromethane, sodium chloride, and sodium borohydride were purchased from SD Fine Chemicals Ltd., Mumbai, India. All the chemicals of analytical grade were used without any further purification. The deionized water (18 MΩ) used in the experiments was obtained from Elga Pure lab Q7/15 water purifier Woodridge, IL, USA.

### 2.1. Synthesis of Polyvinylpyrrolidone (PVP) Capped Silver Nanoparticles

The polyvinylpyrrolidone (PVP) capped silver nanoparticles (Ag-NPs) were synthesized by using two different methods: (i) cold synthesis method and (ii) modified polyol method. In the cold synthesis method, water, sodium borohydride (NaBH_4_), and PVP were used as a solvent, reducing and capping agents, respectively [16]. In this synthesis, 10 mL of 10 mM aqueous solution of silver nitrate (AgNO_3_) was mixed with 10 mL of 0.1 wt.% aqueous solutions of PVP for 30 min in an ice bath condition. The 10 mL of 7 mM aqueous solution of NaBH_4_ maintained at 0 °C was then added dropwise to the solution mentioned above until a yellow color appeared. In the modified polyol method, ethylene glycol and PVP were used as a solvent—reducing and capping agents, respectively [17]. In this synthesis, 10 mL of 0.25 M solution of AgNO_3_ in ethylene glycol was mixed with 10 mL of 0.1 wt.% solutions of PVP in ethylene glycol [17]. The resultant solution was stirred at 120 °C until a yellow color appeared. The solution was allowed to cool down to room temperature (RT) and washed with acetone several times.

### 2.2. Materials Characterization

Various physical techniques were employed for the characterization of PVP-capped Ag-NPs. The X-ray diffraction (XRD) patterns were recorded using the Rigaku MiniFlex X-ray diffractometer (CuKα radiations with λ = 1.541 Å) Austin, TX, USA. Fourier transform infrared (FTIR) spectra of PVP-capped Ag-NPs were collected using a Perkin Elmer spectrometer (resolution: 4 cm^−1^) Akron, OH, USA. The UV-visible absorption spectra of PVP-capped Ag-NPs were analyzed using Perkin Elmer double beam spectrophotometer (Lambda-750, resolution: 0.17–5.00 nm) Akron, OH, USA, having an individual monochromator. The cladding modified fiber’s optical images were captured using a vision measuring machine electronica mechatronic systems, PVT. LTD., India (VMM, Inspec. Vista 2D, Electronica Mechatronic Systems, Pune, India). The morphological studies of silver nanoparticles were carried out using the FEI Nova NanoSEM NPEP303 field emission scanning electron microscope (FE-SEM) at an accelerating voltage of 10 kV.

### 2.3. Sensor Characterization

**Figure 1:**
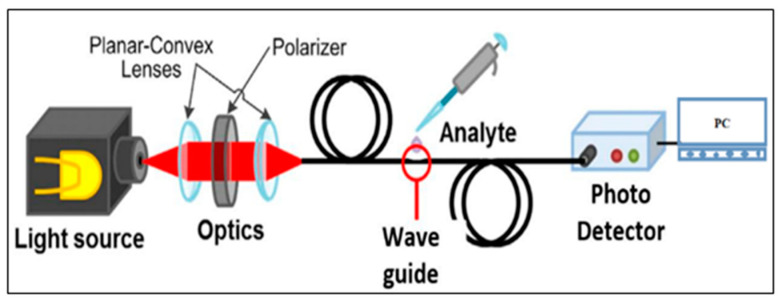
Schematic diagram of plastic optical fiber- (POF) based sensor.

A plastic optical fiber (POF, SH4001) was procured from Mitsubishi Rayon Co., Ltd. (Tokyo, Japan). The plastic optical fiber consists of a polymethyl methacrylate (PMMA) core of 980 μm diameter and 20 μm thick fluorinated polymer cladding. A 1.2 mm thick black polyethylene jacket protected the POF from the external environment. The refractive index values for core and cladding were 1.492 and 1.417, respectively, as given in the datasheet. The light was passed through the fiber and collected using the receiver. The three different colored LEDs were used as light sources, viz., IF E93, IF E96E, and IF E92, with 645, 522, and 470 nm peak emission wavelengths of the red, green, and blue LEDs, respectively. One type of receiver collected light from the three LEDs sources. IF D92 NPN phototransistor with optical response ranging from 400 to 1100 nm was used as the receiver. A Keithley source meter (USA 2400) was used to record the readings of the receiver. Figure 1 shows the schematic diagram of the optical fiber sensor arrangement.

The sensor system comes in a compact box with special-purpose fiber housing and a touch screen user interface that displays the data. The interface also displays the start and stop buttons for three LEDs separately. Both ends of the fiber were polished using emery polish paper for the POF-based sensor fabrication. After optimization experiments, the 10 mm length cladding of the unjacketed portion was removed with dichloromethane. Using the drop-casting method, a 1 μL drop of PVP-capped Ag-NPs was deposited on the removed part of the POF cladding. Then, a 1 µL drop of the sample to be analyzed (aqueous ammonia) was placed on the exposed active part, and changes in output voltage were recorded. For every cycle, the output voltage was initially allowed to become stable (approximately at 2.5 V); after that, the readings were recorded. Finally, an analyte drop was placed onto the exposed part, and changes in output voltage were recorded. The pure deionized water was introduced onto the active fiber part to confirm that the change in output voltage was only due to aqueous ammonia, and reference data were recorded. There was no change in voltage observed for the reference. The performance of PVP-capped Ag-NPs deposited POF-based sensor was recorded at different molar concentrations: 0.054 M, 0.54 M, 5.4 M, and 13.4 M of aqueous ammonia. To understand the selective sensing nature of the POF-based sensor, changes in voltage for potassium and sodium ions were also recorded. The 0.054 M solutions of potassium and sodium ions were prepared by dissolving potassium chloride and sodium chloride in deionized water. A 1 µL drop of each solution (K^+^, and Na^+^) was placed on the POF’s active part (PVP-capped Ag-NP deposited un-cladding).

## 3. Results and Discussion

### 3.1. XRD and FTIR Analysis

**Figure 2:**
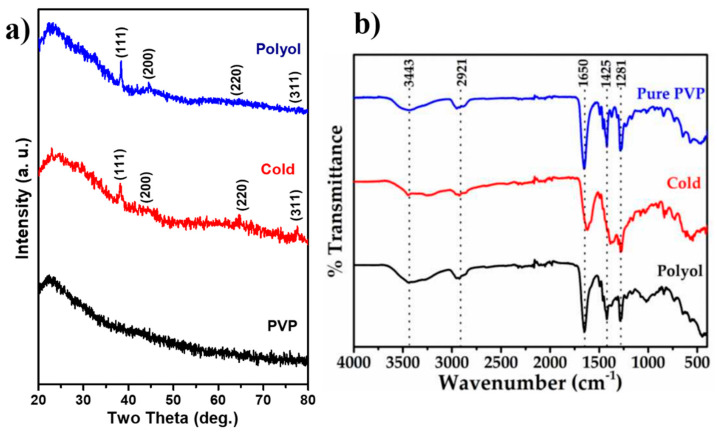
(**a**) XRD patterns for pure PVP, PVP capped Ag-NPs synthesized by using cold synthesis route, and PVP capped Ag-NPs synthesized by using polyol synthesis method, and (**b**) FTIR spectra for PVP, PVP capped Ag-NPs synthesized using polyol method, and PVP capped Ag-NPs synthesized using cold synthesis route.

The XRD patterns for pure PVP, PVP capped Ag-NPs synthesized using a cold synthesis route, and polyol synthesis methods are shown in Figure 2a. The XRD pattern of the samples was obtained at room temperature in the range of 20°–80° with a scan rate of 0.05°, and an X-ray of wavelength 1.5418 Å was used. In all XRD patterns, a broad hump around 2θ = 23° was observed [18]. This is due to the PVP. The XRD pattern for Ag NPs powder prepared using the cold synthesis method shows the diffraction peaks having weak–medium intensities at 38.50°, 44.75°, 64.80°, and 77.70° (2θ values), which correspond to the (111), (200), (220), and (311) planes of metallic silver (Ag), respectively, with FCC crystal symmetry (JCPDS card no. 04-0783) [19,20]. The crystallite size was obtained using the Debye-Scherrer formula as in Equation (1).

(1) $$D=\frac{0.9\lambda }{\beta \cos\theta }$$

where D = crystallite size, λ = X-ray wavelength (1.5418 Å), β = full width at half maximum (FWHM) of (111) plane, and θ = Bragg’s diffraction angle.

The inter-planar spacing (d) and FWHM for (111) plane (2θ = 38.50°) were measured to be 2.35 nm and 1.25°, respectively, for the cold synthesis method derived Ag NPs. The crystallite size estimated for these Ag NPs using the Debye-Scherrer formula is 26.66 nm. The XRD pattern for Ag NPs prepared via the polyol method shows the diffraction peaks having weak-medium intensities at 2θ values of 38.37°, 44.51°, 64.55°, and 77.86°, which correspond to the (111), (200), (220), and (311) planes of metallic Ag having FCC crystal symmetry (JCPDS card no. 04-0783), respectively [19,20]. The inter-planar spacing (d) value and FWHM for (111) plane at 2θ = 38.37° were measured to be 2.34 nm and 3.6°, respectively, for polyol method-derived Ag NPs. The crystallite size obtained for these Ag NPs using the Debye-Scherrer formula was 24.41 nm. The FWHM value of the polyol synthesized Ag NPs was higher than the cold synthesis method. Hence, the particle size of polyol synthesized Ag NPs is smaller than the cold synthesized. These differences in Ag NPs size and FWHM might be due to the PVP molecule’s attachment to Ag ions during both synthesis methods. More PVP interacts with Ag ions during polyol synthesis leading to a smaller size of the nanoparticles. At the same time, less PVP interacts with Ag ions during cold synthesis because of the presence of water molecules leading to the formation of larger nanoparticles [21]. The FTIR spectra of pure PVP, PVP-capped Ag-NPs synthesized via polyol, and cold synthesis methods are shown in Figure 2b. For PVP capped Ag-NPs synthesized via polyol method, the FTIR peaks at 3443 cm^−1^, 2921 cm^−1^, 1385 cm^−1^, and 1281 cm^−1^ represent N-H stretching in the amine group, C-H stretching in the alkene group, NO_3_ functional group, and the N-H-O group, respectively. These are the functional groups present in the PVP molecule. The C-N bond’s asymmetric and symmetric stretching peaks were shifted from 1095 cm^−1^ and 1070 cm^−1^ to 1167 cm^−1^ and 1075 cm^−1^, respectively. Furthermore, the FTIR peak at 1018 cm^−1^ is broad, indicating coordinative chemical bonding of the N atom of PVP to the Ag surface [22]. Further, the vibrational FTIR peak of the carbonyl group (C=O) was also noted to have shifted from 1622 cm^−1^ to 1648 cm^−1^. The shifting of these FTIR peaks indicates that the N and O elements of the PVP molecule have adhered to the surface of Ag-NPs [23]. The PVP-capped Ag-NPs synthesized via the cold synthesis method also show similar FTIR peaks with slight broadening. This broadening may be due to the hydrogen-bonding interaction in PVP-capped Ag NPs [24]. In addition, the FTIR peak at 1018 cm^−1^ corresponding to bonding between PVP and Ag-NPs is entirely missing in cold method synthesized particles, which may be due to PVP additionally binding with water, and therefore, there is not enough PVP available for the bonding with reduced Ag ions [22]. However, in the polyol method, all the PVP bonds well with reduced Ag ions.

### 3.2. UV-Visible Analysis

**Figure 3:**
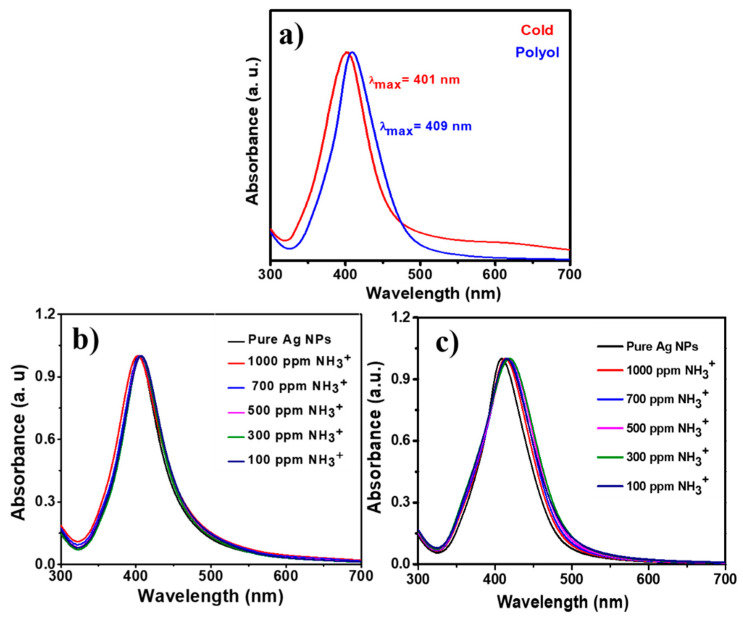
(**a**) UV-visible absorption spectra for PVP capped Ag-NPs synthesized, UV-visible absorption spectra of PVP capped Ag-NPs synthesized by using (**b**) cold and (**c**) polyol synthesis methods with varying concentrations of ammonium solution.

The UV-visible spectra for the PVP-capped Ag-NPs synthesized via cold and polyol synthesis are shown in Figure 3a, respectively. It is evident from the absorption spectra that the absorbance peak is narrower for the cold synthesis method derived from PVP-capped Ag-NPs than for the polyol method. The values for λ_max_ (SPR) and FWHM were measured to be 401 and 69 nm, respectively, for PVP capped Ag-NPs synthesized via the cold synthesis method. Whereas for the polyol synthesis method, the values of λ_max_ and FWHM were noted to be 409 and 66 nm, respectively. These differences in the FWHM and λ_max_ values may be due to the strong reducing agent NaBH_4_ in cold synthesis, leading to a fast reaction. At the same time, ethylene glycol is a weaker reducing agent used in polyol synthesis resulting in a slower reaction. The weaker reducing agent results in the formation of poly-dispersed nanoparticles during the polyol synthesis method. A stronger reducing agent of monodispersed nanoparticles forms during the relatively fast cold synthesis method [25]. The UV-visible absorption spectra of PVP capped Ag-NPs synthesized via (a) cold and (b) polyol synthesis methods are shown in Figure 3b,c at different concentrations (50–1000 ppm) of ammonium solution addition during both synthesis methods, respectively. As reported previously, adding ammonia in a small quantity leads to red-shift/long-wavelength SPR changes [17]. In these spectra, the absorbance peaks are redshifted to the longer wavelengths upon adding ammonium ions. This is due to the formation of Ag(NH_3_)^2+^ complex and aggregation of nanoparticles. For the polyol method synthesized Ag NPs [Figure 3c], intensity changes are observed at the longer 480 nm wavelength. Hence, an absorption ratio A_1_/A_2_ is calculated to track these changes, where A_1_ is the intensity of the peak at 480 nm and A_2_ is the intensity of the peak at 409 nm. It was observed that this absorption ratio decreased with the increasing concentration of ammonium solution.

### 3.3. Optical Image Analysis

**Figure 4:**
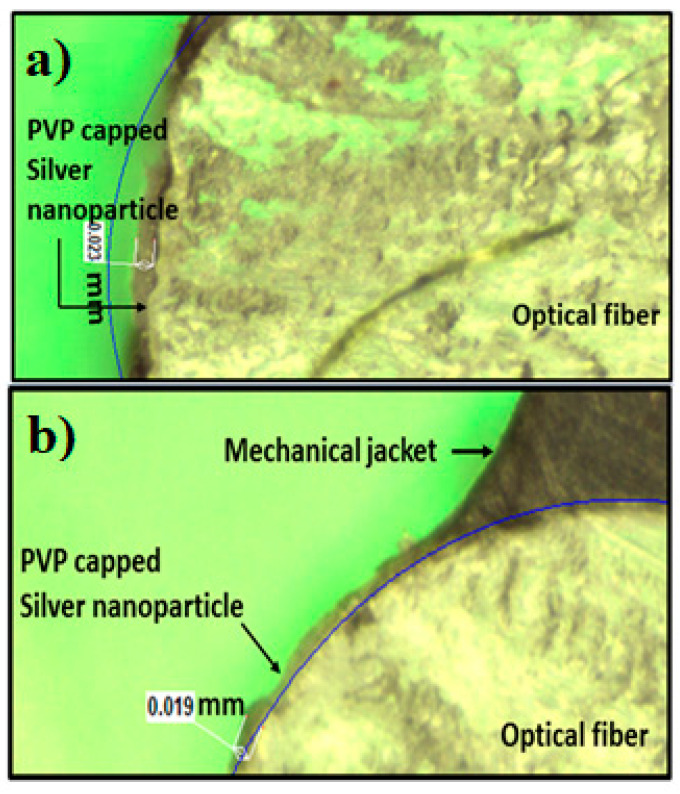
Optical images for cladding modified POF surfaces coated with PVP capped Ag-NPs synthesized by using (**a**) cold and (**b**) polyol synthesis methods.

The optical images for cladding modified POF surfaces coated with PVP-capped Ag-NPs synthesized via cold and polyol synthesis methods are shown in Figure 4a,b, respectively. In both cases, the thickness of as-deposited PVP capped Ag-NPs on the POF surface was nearly the same, demonstrating that almost an equal volume of cold and polyol synthesized PVP-capped Ag-NPs was deposited on the unclassed part of the fiber. The average thickness values of PVP-capped Ag-NPs on the POF surface are 23 μm and 19 μm for cold and polyol synthesized, respectively.

### 3.4. FESEM Image Analysis

**Figure 5:**
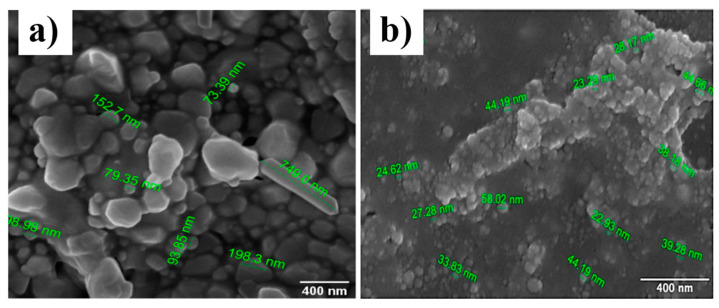
(**a**) FESEM image of silver nanoparticles synthesized using polyol method and (**b**) FESEM image of silver nanoparticles synthesized using cold method.

Figure 5a,b shows the FESEM images of drop-casted thin films of silver nanoparticles synthesized using the polyol and cold synthesis methods, respectively. Both images were taken at the same magnification of 200,000×. Figure 5a reveals the formation of spherical and cylindrical nanoparticles. Whereas in Figure 5b, we can see only the formation of spherical nanoparticles with aggregation of some nanoparticles. While preparing the thin films using the drop-casting method, we heated the thin films on a hot plate at 60 °C to quickly dry up the deposited layers of silver nanoparticles. This heating of silver nanoparticles has caused the silver nanoparticles to be aggregated in some places. This implies that the aggregation of silver nanoparticles seen in the images is because of the heating of silver nanoparticles and not because of the synthesis procedure.

As seen in the images, the polyol synthesis method has resulted in spherical and cylindrical nanoparticles. On the other hand, the cold synthesis method has resulted in only spherical nanoparticles. The formation of cylindrical nanoparticles in the polyol synthesis method might explain the better results obtained for ammonium detection using the polyol synthesized product because, according to the literature review, cylindrical nanoparticles show the highest sensitivity for SPR-based detection [26].

### 3.5. Ammonium Sensing Study

**Figure 6:**
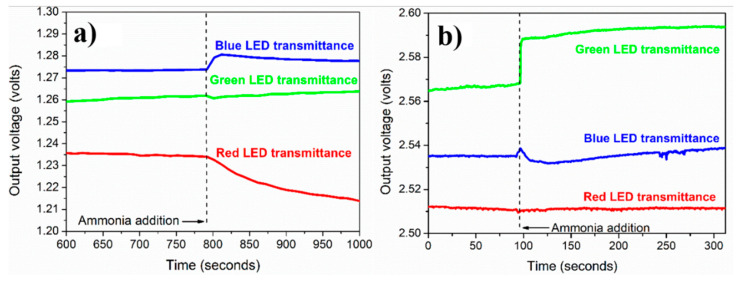
Variation of POF output voltage with time for PVP capped Ag-NPs synthesized by using (**a**) cold and (**b**) polyol synthesis methods (ammonium solution concentration = 2 M).

Figure 6a,b shows the variation of POF output voltage with time for PVP-capped Ag-NPs synthesized via (a) cold and (b) polyol synthesis methods, respectively. This POF response was recorded at a 2 M concentration of ammonium solution for all three LEDs: red, blue, and green. From Figure 6, it was observed that the responses for blue and green LEDs are better than for red LED in both synthesis method prepared Ag NPs. Further, in the case of POF coated with PVP capped Ag-NPs synthesized via polyol method, all three LEDs’ responses are better, almost double that of the POF coated with PVP capped Ag-NPs synthesized via cold synthesis method. For PVP capped Ag-NPs synthesized via cold synthesis method, the output voltage was arbitrarily set in the range 1.24 V–1.28 V [Figure 6a], whereas, for PVP capped Ag-NPs synthesized via polyol synthesis method, the output voltage was set in the range 2.50–2.58 V [Figure 6b]. This output voltage merely depends upon the placement of the optical fiber in the fiber-housing and has almost no significance on the changes in output voltage. Finally, voltage changes after adding aqueous ammonia are considered, not the initial arbitrary output voltage.

The output voltage for the cold synthesis method is the highest for the blue LED, as observed in Figure 6a. This was expected because ammonium addiction causes changes in the λ_max_ (~400 nm) of the UV-visible absorption spectrum. Hence, the blue LED was selected for all further experiments dealing with cold route synthesized PVP-capped Ag-NPs. For polyol synthesized PVP-capped Ag-NPs (Figure 6b), the red LED light output voltage was not affected by ammonium addition. In contrast, the output voltage of blue and green LEDs changed in response to ammonium addition. The green LED gives the highest and most stable response, and the blue LED response dies out after some time. The blue LED was expected to respond to ammonium addition. However, a better response was obtained for the green LED, probably due to the absorption ratio A_1_/A_2_ observed in the visible absorption spectrum, which revealed the long-wavelength region of 480 nm, which corresponds to the bluish-green color as observed in Figure 6b. Since the green LED response was much better than others, all further experiments were performed using the green LED as the light source.

Since the response for the green LED using polyol route synthesized silver nanoparticles is the best, we will use the corresponding output voltage changes to define the response time. Figure 6b shows that it takes about 5 s for the output voltage to increase and saturate after ammonium addition. Hence, the optical fiber system’s response time (or sensing speed) can take 5 s.

### 3.6. Sensing Repeatability, Calibration Curves, and Selectivity

**Figure 7:**
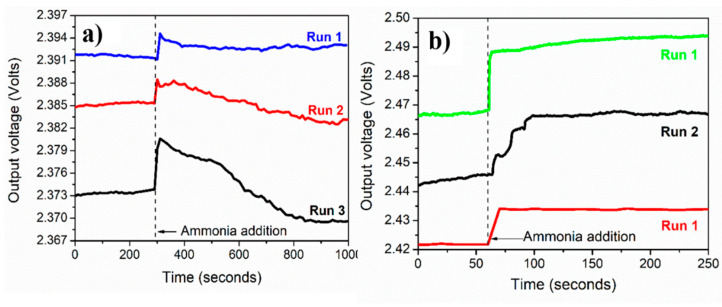
Repeatability: variation of output voltage with time for PVP capped Ag-NPs synthesized by using (**a**) cold and (**b**) polyol synthesis methods (recorded for 3 cycles and at ammonium solution concentration = 2 M).

The ammonium sensing repeatability was studied for both PVP-capped Ag-NPs synthesized via cold and polyol synthesis methods, as shown in Figure 7a,b for 3 runs each. The repeatability curves were recorded using: (i) the blue LED for cold method synthesized PVP capped Ag-NPs, and (ii) green LED for the polyol method synthesized PVP capped Ag-NPs. The standard deviation values for the green and blue LEDs were found to be 0.013 and 0.0012, respectively.

**Figure 8:**
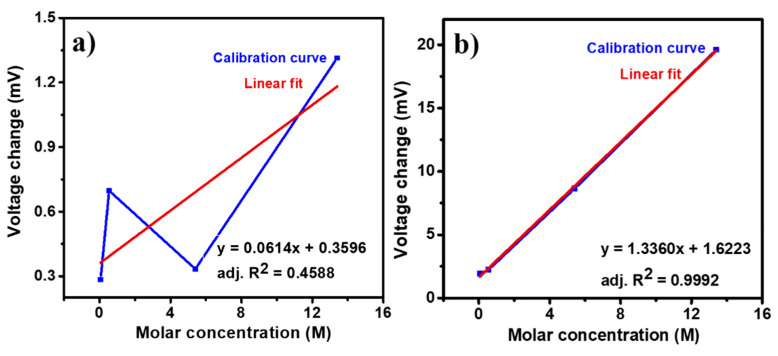
Calibration curve: variation of voltage changes with molar concentration of ammonium solution and linear fit to the voltage change for PVP capped Ag-NPs synthesized by using (**a**) cold and (**b**) polyol synthesis methods.

These lower values of standard deviation indicate good repeatability of the sensing properties of PVP-capped Ag-NPs-coated un-cladded POF-based ammonium sensor. Figure 8a,b shows the change in voltage for varying ammonium ions molar concentrations (0.054 M, 0.54 M, 5.4 M, and 13.4 M) for PVP-capped Ag-NPs synthesized via cold and polyol synthesis methods, respectively. The voltage change was observed to be proportional to the ammonium solution concentration. A linear fit was used as the calibration curve to obtain a straight line.

**Figure 9:**
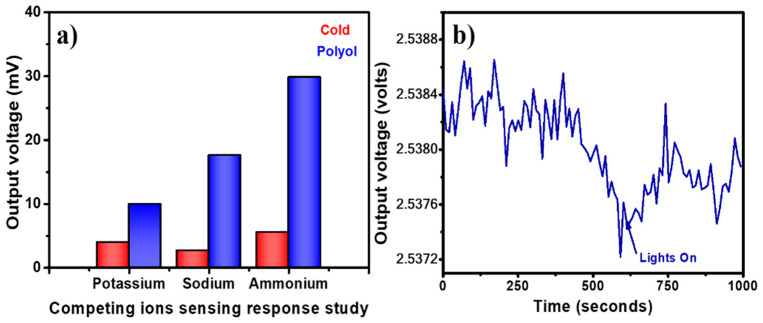
(**a**) Selectivity of Polyol synthesized product and cold synthesized product towards ammonium ion sensing, and (**b**) ambient light effect for PVP capped Ag-NPs synthesized by using polyol method.

For PVP capped Ag-NPs synthesized via the cold synthesis method, the sensor exhibited a linear relationship with R^2^ = 0.4588 (Figure 8a) for 0.054 to 13.4 M is varying molar concentration of ammonium with a detection limit of 159.4 mM and a sensitivity of 0.06 mV/M. For PVP capped Ag-NPs synthesized via polyol synthesis method, the sensor exhibited a linear relationship with R^2^ = 0.9992 (Figure 8b) for 0.054 to 13.4 M varying molar concentration with a detection limit of 48.9 mM and a sensitivity of 1.33 mV/M. The limit of detection of the sensor was calculated using the ratio of thrice the standard deviation to the sensor’s sensitivity near zero concentration. Figure 9a shows the selectivity for PVP-capped Ag-NPs synthesized via cold and polyol. For the selectivity study, blue and green LEDs were used for cold and polyol synthesized PVP-capped Ag-NPs, respectively. The potassium and sodium ions were selected because they are the natural competitors in natural water/soil samples. The sensor showed the highest voltage change for ammonium ions compared to others for PVP-capped Ag-NPs synthesized via both methods. Thus, the present sensor shows excellent selectivity towards ammonium ion sensing. Since it is an optical sensing probe designed for in situ sensing, the effect of ambient/environmental light on the optical fiber must be studied. Figure 9b shows one such experiment for PVP-capped Ag-NPs synthesized via the polyol synthesis method. During an ammonium sensing experiment, laboratory lights were switched off for the first 10 min (600 s) and then switched on for the next 10 min. Switching on the light did not affect the optical fiber sensing readings. This proves that the PVP-capped Ag-NPs coated, un-cladded POF-based ammonium sensor is reliable for open fields for real-time and in situ sensing.

### 3.7. Sensing Mechanism

**Figure 10:**
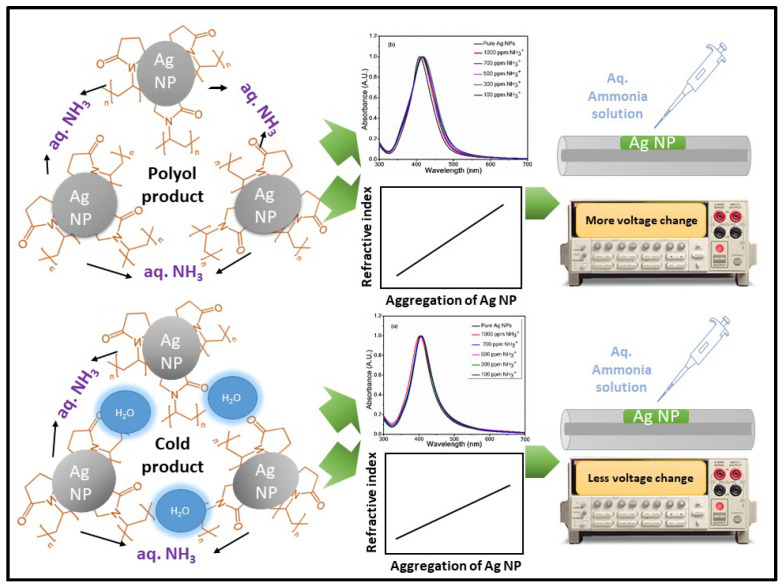
The sensing mechanism of (**a**) cold synthesis method produced PVP capped Ag-NPs coated optical fiber-based ammonium sensor, and (**b**) polyol synthesis method produced PVP capped Ag-NPs coated optical fiber-based ammonium sensor.

The sensing mechanism of the PVP-capped Ag-NPs-coated POF-based ammonium sensor is depicted in Figure 10. The sensing scheme shows that PVP-capped Ag-NPs have negative charge capping due to the vinyl chains. When PVP-capped Ag-NPs are exposed to an ammonium solution, the positively charged ammonium ions bind to negatively charged PVP-capped Ag-NPs. Hence, the aggregation of PVP-capped Ag-NPs increases. This further causes the SPR-based changes in the UV-visible absorption spectrum of the PVP-capped Ag-NPs. The aggregation of nanoparticles also leads to a change in the refractive index of the PVP-capped Ag NPs [27]. The cladding modified optical fiber exposes the evanescent field to the external environment.

The interaction between this evanescent field and the external environment enables us to detect the refractive index of the surrounding medium [28]. Further, the change in refractive index causes more light to be confined in the optical fiber, which leads to the output voltage change as seen in the data recorded by the receiver during the addition/casting of aqueous ammonium solution to the PVP-capped Ag-NPs [28]. In the case of the polyol method synthesized product, PVP-capped Ag-NPs interact with ammonium ions to form aggregates of Ag-NPs, leading to the observed voltage changes in optical fiber upon aqueous ammonia addition. In the case of the cold route synthesized product, PVP capped Ag-NPs bind additionally with the water molecule. Therefore, the PVP-capped Ag-NPs are not readily available for bonding with ammonium ions [22]. This leads to less aggregation of cold route synthesized PVP-capped Ag-NPs upon addition of ammonium ions than the polyol route synthesized PVP-capped Ag-NPs.

### 3.8. Comparison of Our Work with the Literature Review

Much research has been carried out on detecting ammonium using silver nanoparticles. Some of this work has been reported in Table 1. As seen from Table 1, previously reported work shows good detection n limit and sensitivity towards ammonium detection. However, the previously reported works use the colorimetric method for ammonium detection. The colorimetric method is limited to laboratory sensing and cannot be used for on-site detection of ammonium. At the same time, our reported work uses optical fiber-based evanescent wave absorption spectroscopy to detect ammonium. Optical fiber in our work makes the on-site detection of ammonium possible.

## 4. Conclusions

The cold and modified polyol synthesis methods are used to synthesize PVP-capped Ag NPs. The prepared Ag NPs are characterized using XRD, FTIR, UV-visible spectroscopy, and optical microscopy. The characterization studies indicate that Ag-NPs capping with PVP is not more efficient during cold synthesis than the polyol synthesis method. The sensing characteristics, viz., sensing response, repeatability, competing for interference, and the ambient light effect, are studied for PVP capped Ag-NPs modified optical fiber-based sensor. The polyol method synthesized PVP-capped Ag-NPs has a detection limit of 48.9 mM with a good linear relationship (R^2^ = 0.9992) between voltage and ammonium ion concentration in the range of 0.054 to 13.4 M. The cold method synthesized PVP-Ag-NPs has a detection limit of 159.4 mM with a poor linear relationship (R^2^ = 0.4588) between voltage and ammonium ion concentration in the range of 0.054 to 13.4 M. The polyol method synthesized PVP capped Ag-NPs showed better sensitivity of 1.33 mV/M compared to the sensitivity of 0.06 mV/M compared to the sensitivity of 0.06 mV/M shown by the synthesized cold method. The polyol method synthesized PVP-capped Ag-NPs has a better aqueous ammonium sensing performance. This might be due to inefficient PVP binding in the cold synthesis method because of the presence of water in the synthesis process compared to the polyol synthesis method. A rapid, cost-effective, and portable aqueous ammonium sensing approach is developed using POF-based sensors.

## Figures and Tables

**Table 1:** Reports of some studies on ammonium sensing using silver nanoparticles.

Sr. No.	Materials Used	Detection Range	Sensitivity	Detection Principle	Reference
1.	Silver nanoparticles synthesized using tannic acid	0–500 ppm	3.5/200 ppm	Colorimetric	[29]
2.	Silver nanoparticles synthesized using durian fruit shell	500–3000 ppm	15/3000 ppm	Colorimetric	[30]
3.	Silver nanoparticles/Polyaniline composite thin films	0–800 mM	0.2038 mM	Colorimetric	[31]
4.	Silver nanoparticles synthesized using the polyol synthesis method and cold synthesis method	0.054–13.4 M	1.33 mV/M	Optical fiber evanescent absorption spectroscopy	Present work

## Data Availability

The data presented in this study are available on request to the corresponding author.
